# Multinozzle Emitter for Improved Negative Mode Analysis of Reduced Native *N*-Glycans by Microflow Porous Graphitized Carbon Liquid Chromatography Mass Spectrometry

**DOI:** 10.1021/acs.analchem.3c03649

**Published:** 2024-04-01

**Authors:** Melinda Wojtkiewicz, Sabarinath Peruvemba Subramanian, Rebekah L. Gundry

**Affiliations:** † CardiOmics Program, Center for Heart and Vascular Research, and Department of Cellular and Integrative Physiology, University of Nebraska Medical Center, Omaha, Nebraska 68198, United States.

## Abstract

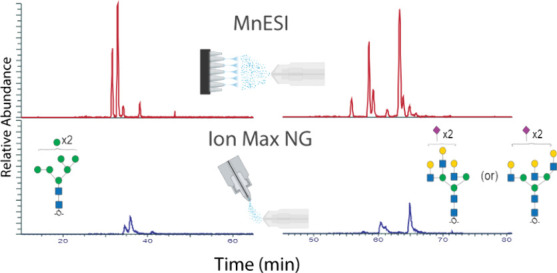

Microflow porous graphitized carbon liquid chromatography (PGC-LC) combined with negative mode ionization mass spectrometry (MS) provides high resolution separation and identification of reduced native *N*-glycan structural isomers. However, insufficient spray quality and low ionization efficiency of *N*-glycans present challenges for negative mode electrospray. Here, we evaluated the performance of a recently developed multinozzle electrospray source (MnESI) and accompanying M3 emitter for microflow PGC-LC-MS analysis of *N*-glycans in negative mode. In comparison to a standard electrospray ionization source, the MnESI with an M3 emitter improves signal intensity, identification, quantification, and resolution of structural isomers to accommodate low-input samples.

Mass spectrometry (MS)-based strategies for released *N-*glycan analysis include those that analyze derivatized glycans (e.g., glycans labeled by permethylation, procainamide, 2-aminobenzoic acid, or 2-aminobenzamide acid) or reduced native glycans.^1−7^ Derivatization is used to improve ionization and, consequently, enhance detection and to improve chromatographic separation by eliminating anomericity on the reducing end of the glycan.^2,3^ However, chemical modifications on glycans (e.g., phosphorylation, acetylation) are lost during permethylation,^5,8,9^ and labeling can be expensive for high-throughput applications. Reduced native *N*-glycan analysis includes a reduction step to eliminate anomericity but otherwise avoids chemical derivatization.^4^ Consequently, reduced native *N*-glycans require a simpler, cost-effective sample preparation process that preserves glycan modifications.^10^

When using liquid chromatography MS (LC-MS) to analyze reduced native *N*-glycans, porous graphitized carbon (PGC) stationary phase provides a higher resolution of structural isomers compared to hydrophilic interaction^11,12^ and negative ion mode generally provides more structural information than positive mode due to producing more fragments by cross-ring cleavage than glycosidic cleavage, which yields structure-specific diagnostic ions.^13−17^ However, widespread implementation of nanoflow PGC-LC-MS has been limited by common operational challenges with performing nanoflow MS in high-throughput settings^18^ and difficulty in manually preparing or obtaining commercial PGC columns.^19,20^ Therefore, microflow PGC-LC-MS is commonly used for reduced native *N*-glycans due to its robustness and ease of column generation, despite it being less sensitive than nanoflow. However, insufficient spray quality and the low ionization potential of *N*-glycans present challenges in negative mode electrospray analyses. Here, we evaluated the performance of a recently developed multinozzle electrospray source (MnESI) and accompanying M3 emitter for microflow PGC-LC-MS analysis of complex biological samples of reduced native *N*-glycans in negative mode. In comparison to a standard electrospray source, which is commonly used for microflow applications, the MnESI with M3 emitter improves signal intensity, detection, quantification, and resolution of isomers to accommodate low-input samples.

## Experimental Section

**Figure 1:**
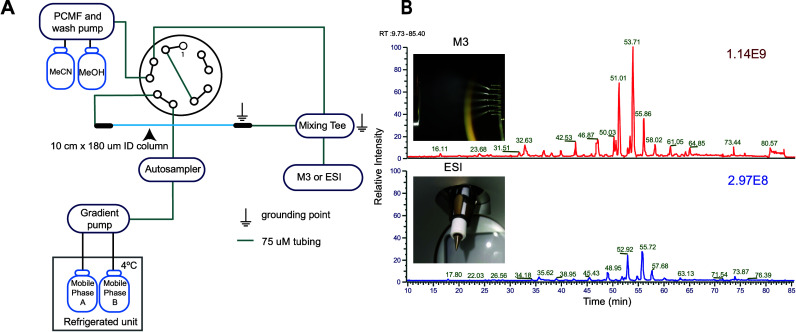
Overview of LC-MS setup and total ion chromatograms (TIC) using the MnESI source with the M3 emitter and the Ion Max NG ion source with the ESI emitter. (A) Plumbing diagram of LC to MS source. (B) Images of M3 and ESI emitters and globally normalized representative TIC obtained from the analysis of undiluted sample using each emitter type. PCMF = postcolumn makeup flow.

*N*-Glycans were prepared from human serum (NIST reference standard 909c) using the glyPAQ kit (beta version; ProtiFi, Fairport, NY) per manufacturer’s instructions. Five μL of sample (equivalent to *N*-glycans released from 40, 4, 0.4, 0.04, and 0.004 μg total serum protein for neat and diluted samples, respectively) were injected onto an Ultimate 3000 UHPLC system capillary pump coupled to an Orbitrap Eclipse mass spectrometer (ThermoFisher Scientific, Waltham, MA). The microcolumn (180 μm × 100 mm) was packed in house with Hypercarb 3 μm PGC (ThermoFisher Scientific)). The column flow was 2 μL/min, and a postcolumn makeup flow of acetonitrile at 3 μL/min was connected with a tee junction (Figure 1A).^21,22^ Ionization was achieved using either the electrospray probe (H-ESI probe with no heated auxiliary gas) with a low flow needle on the Ion Max NG ion source (ThermoFisher Scientific) or MnESI source (Newomics, Berkely, CA) with a Microfabricated Monolithic Multinozzle (M3) 5-nozzle emitter. Skyline-daily (64-bit) 21.1.1.223^23^ and GlycoWorkBench v2.1^24^ were used for structural analyses and GlycReSoft version 0.4.13^25^ was used for compositional analyses. Structures were identified based on composition, B/Y- and C/Z-ions observed in MS/MS fragmentation, and order of elution^26^ using an in-house library. Glycan identity was mapped using GlyToucan accession and Glyconnect glycompozitor using composition as the query.^27^ Diagnostic ions used for glycan identification are provided in Supporting Information, Table S1. Statistical analysis was performed using GraphPad Prism 10.0.0. Methodological details are listed in Supporting Information, Methods.

## Results and Discussion

### Improved *N*-Glycan Detection with MnESI Source and M3 Emitter

**Figure 2:**
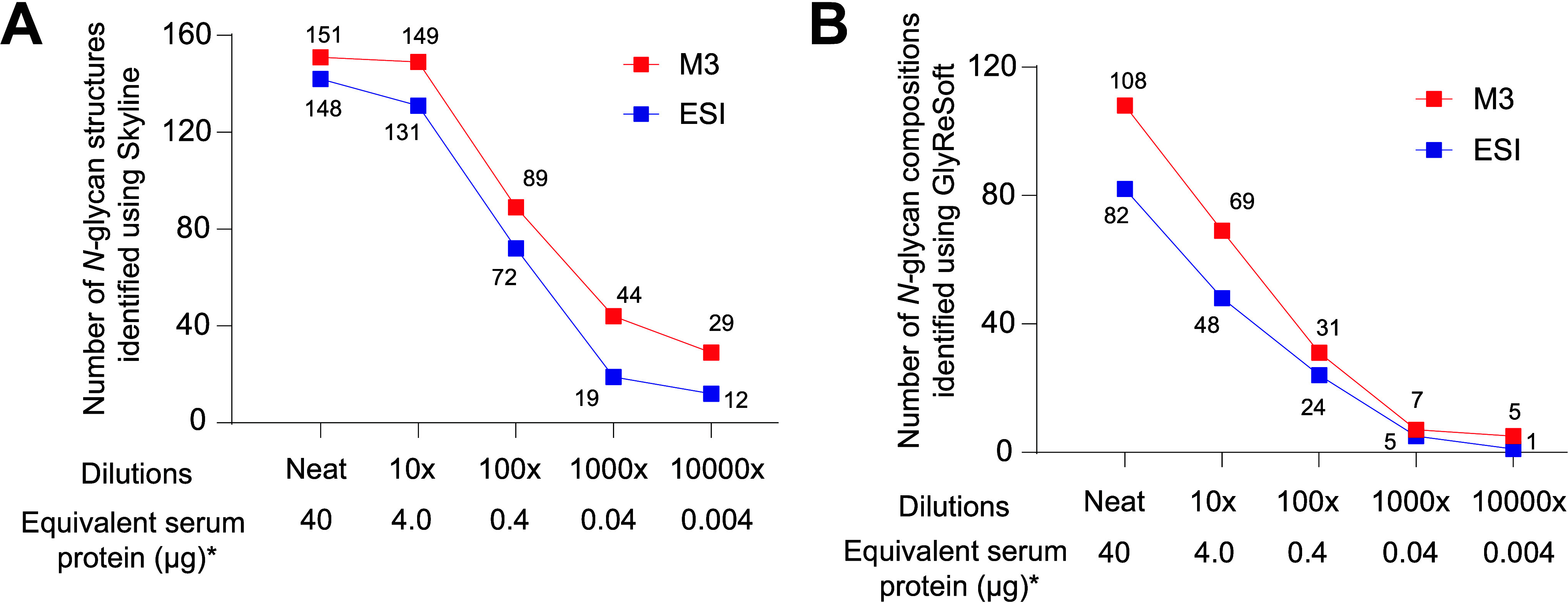
Overview of results showing improved detection of *N*-glycans using the MnESI source with M3 emitter compared to the Ion Max NG ion source with ESI emitter. (A, B) Average number of *N*-glycan structures (A) and compositions (B) detected across three injections of each dilution using the M3 or ESI emitter.

The M3 emitter splits the microflow input into five nanospray plumes, whereas the ESI emitter provides a single plume (Figure 1B). This results in a nearly 4-fold increase in relative intensity in the total ion chromatogram (TIC; Figure 1B) and increased detection of *N*-glycan structures for the M3 compared to the ESI (Figure 2A). On average, for neat samples, the ESI emitter resulted in the detection of 148 *N*-glycan structural isomers, while the M3 resulted in 151 *N*-glycan isomers. For more dilute samples, the M3 emitter resulted in an average of 13%, 23%, 131%, and 141% increase in *N*-glycan structures for each dilution tested (10× to 10000×). The structural assignments were generated using Skyline. However, Skyline has been used in relatively few glycan studies compared to composition-based software, in part due to the limited availability of the necessary structure library files.^28−32^ Thus, we also analyzed data for glycan compositions using GlycReSoft,^25^ as this may benefit more investigators. GlycReSoft identified 82 and 108 compositions when the ESI and M3 emitters, respectively, were used for undiluted samples (Figure 2B).

The increase in compositions identified using the M3 compared to the ESI emitter was consistently above 29% for all dilutions tested. Extracted ion chromatograms of three examples of *N*-glycans preferentially detected by the M3 emitter demonstrate the improvement for both nonsialylated and sialylated glycans (Supporting Information, Figure S1). Raw area counts of annotated glycan peaks observed in three technical replicates are provided in Supporting Information, Figure S2 and Table S2. Overall, whether performing structure or composition analysis, the MnESI source with the M3 emitter yields more *N*-glycan identifications than the Ion Max NG ion source with the ESI emitter.

### Improved Postcolumn Resolution of Glycan Isomers with an MnESI Source and M3 Emitter.

**Figure 3:**
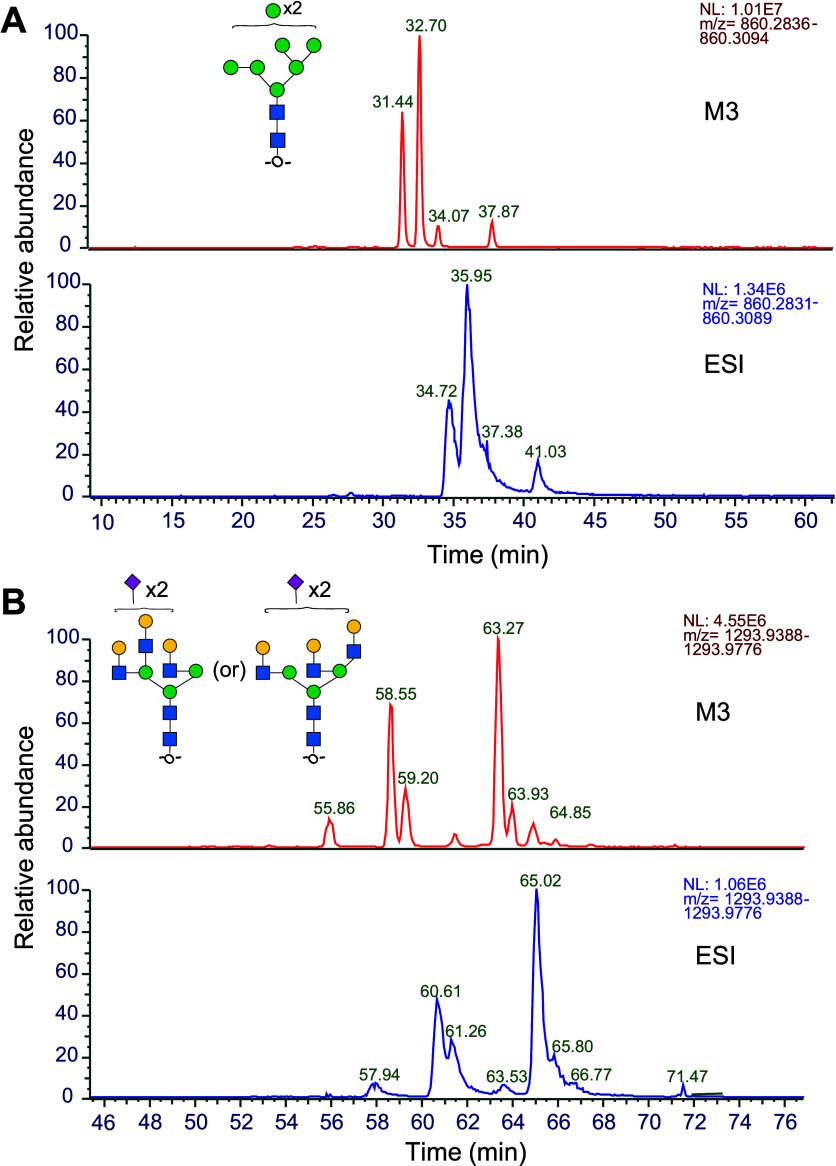
Extracted ion chromatograms (XIC) for two glycan masses showing improvement in peak separation and baseline resolution of structural isomers in chromatograms obtained using MnESI with an M3 emitter compared to the Ion Max NG ion source with an ESI emitter. (A) XIC of HexNAc2Hex8: total of 4 isomers (3 isomers of Man8 and Man7Glc1) were resolved with MnESI. (B) XIC of HexNAc5Hex6NeuAc2: Multiple isomers of complex triantennary structure with two LacNAc on 3-Man or 6-Man arm and disialylated (3,3- or 3,6- or 6,6-linked) were resolved with MnESI. Note: Structure and linkage assignments for proposed structures need to be confirmed through orthogonal approaches, with proposed isomers shown here.

We observed improved resolution for *N*-glycan structural isomers when using M3 compared to ESI (Figure 3). Complex tri- and tetra-antennary glycans exist in multiple isomeric forms, often as sialylated or fuco-sialylated structures. Ionizing sialylated glycans in the negative mode is a known challenge. Therefore, we expect the relative increases in complex tri- and tetra-antennary glycans are due to better resolution and ionization of glycans achieved in the M3 source compared to ESI. This improvement was observed across the chromatogram for different structure types and not limited to a specific elution segment or glycan class. The observed improvement in resolution varies among glycans, and extracted ion chromatograms showing examples from other glycan classes are provided in Supporting Information, Figure S3.

The only difference in these experiments was the ionization source (i.e., all other aspects of plumbing were identical). We speculate that the improvement may be due to glycan–metal interactions that occur in the ESI emitter (low flow metal needle is 15 cm) leading to adsorption that causes poor peak shape, tailing, and reduced recovery, which is minimized when using the silicon-based M3 emitter.^33,34^ Another possibility is an additional volume within the ESI emitter (294 nL) that would cause postcolumn mixing and is avoided in the M3 (12 nL). Neither hypothesis can be tested, however, as neither emitter is available in opposing materials.

### Improved Performance for Quantification of Low Abundance *N*-Glycans with an MnESI Source and M3 Emitter

**Figure 4:**
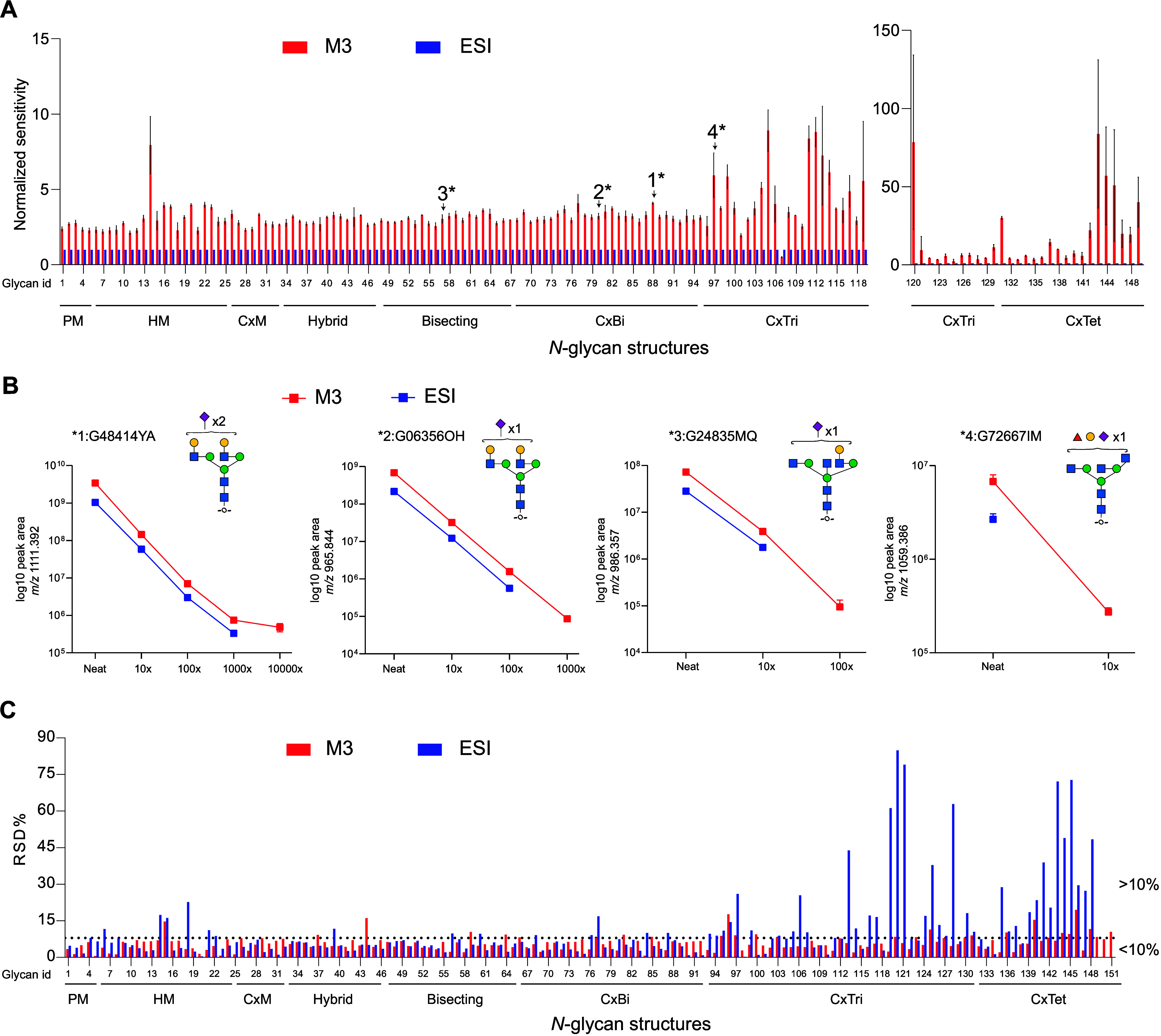
Improvements to *N-*glycan detection and reproducibility when using the MnESI source with M3 emitter compared to the Ion Max NG ion source with ESI emitter. (A) Enhanced detection of all *N*-glycan structures displayed as peak area observed using M3 normalized to ESI. Arrows indicate bars for *N*-glycans shown in detail in panel B and data are three technical replicates. (B) Log 10 peak area for four representative *N*-glycans plotted over the sample dilution series including an error bar for standard deviation. (C) Percent relative standard deviation (RSD%) of peak areas for all *N*-glycan structures detected. Abbreviations: PM - paucimannose, HM - high-mannose, CxM - complex monoantennary, CxBi - complex biantennary, CxTri - complex triantennary, CxTet - complex tetraantennary

Accurate relative quantification of *N*-glycans depends on the ability to resolve structures, the linear range of detection, and the repeatability in peak area or intensity. Overall, in comparison to the ESI, the M3 emitter results in a higher peak area for all *N*-glycan classes detected (Figure 4A). The increase in peak area ranged from 2% to 75%, with the highest increase observed for complex triantennary and tetrantennary structures. This increase in the peak area impacts the linear range of detection (Figure 4B). For higher abundance *N*-glycans (G48414YA, G06356OH), a similar linear range is observed for M3 and ESI emitters. However, for lower abundance *N*-glycans, the difference in detection and linear range is more significant (G24835MQ, G72667IM; Figure 4B). The M3 emitter also led to a significant reduction in percent relative standard deviations of peak area compared with the ESI (Figure 4C). This trend was observed for all *N*-glycan classes, and like the effect on abundance, a more dramatic improvement in repeatability was observed for complex tri-antennary and tetra-antennary structures.

Overall, the improvements to the linear range of detection and repeatability provide evidence that the M3 emitter will benefit future quantitative studies of *N*-glycans, especially from low abundance samples. Relevant for this, we observed that the M3 emitter repeatedly improves peak areas for all glycans, but the relative increase is variable among different glycans within a sample, and complex tri-antennary and tetra-antennary experience a greater relative increase in peak area compared to other classes. We expect this will influence interpretation of results of methods that use the peak area of a glycan/total peak area for all glycans (i.e., total area normalization). Simply, data acquired using the M3 emitter would not replicate those of the ESI emitter if peak area of a glycan was compared as a percentage of the total glycan area. Consequently, alternative data normalization methods may be more appropriate when aiming to compare data among studies, as previously suggested.^35,36^

## Conclusions

In comparison to the Ion Max NG ion source with a low-flow ESI emitter, the MnESI source with an M3 emitter improves signal intensity, identification, and resolution of structural isomers when analyzing reduced native *N*-glycans in negative mode by microflow PGC-LC-MS. These improvements were observed across all *N*-glycan compositions and structures detected.

While all compositions can be represented by multiple isomers, identification of complex tri-antennary and tetra-antennary structures is especially complicated due to the presence of a higher number of possible isomers. This results in overlapping peaks that are difficult to resolve, identify, and quantify. Therefore, the improved resolution achieved with the M3 emitter resulted in an improved ability to identify and quantify these complex structures compared to ESI. In addition to enhanced detection, improvements in signal intensity and repeatability were also observed, suggesting that the MnESI source will benefit quantitative analyses.

While other spray methods have been found to improve ionization of oligosaccharides, to our knowledge vibrating sharp-edge spray ionization has been limited to purified mono-, di-, or trisaccharides^37−41^ and does not provide additional separation, and subambient pressure ionization with nanoelectrospray has been applied for positive mode analysis of complex biological glycan samples.^42^ Overall, given the ease of installation and performance improvements, the MnESI provides unique advantages for the negative mode analysis of complex biological glycans.

## Supporting Information Available

Supporting Methods and Figures S1–S3 (PDF)
Table S1: Diagnostic ions and relative retention times used for identifying glycan compositions and structures; Table S2: Normalized peak area and percentage relative standard deviation (RSD%) from peak area obtained from Skyline analysis (XLSX)
