# Supplementary material for: Multinozzle Emitter for Improved Negative Mode Analysis of Reduced Native N-Glycans by Microflow Porous Graphitized Carbon Liquid Chromatography Mass Spectrometry
Source: Anal Chem. 2024 Apr 1;96(15):5746–51. doi: 10.1021/acs.analchem.3c03649 (PMC11024887; doi:10.1021/acs.analchem.3c03649)
Supplement: Supplementary file 1 — ac3c03649_si_001.pdf [file ac3c03649_si_001.pdf]

Multi-nozzle emitter for improved negative mode analysis of reduced native *N*-glycans by microflow porous graphitized carbon liquid chromatography mass spectrometry

Melinda Wojtkiewicz[a¥], Sabarinath Peruvemba Subramanian[a¥], and Rebekah L. Gundry[a*]

[a]CardiOmics Program, Center for Heart and Vascular Research, and Department of Cellular and Integrative Physiology, University of Nebraska Medical Center, Omaha, NE, 68198, USA.
*Email: rebekah.gundry@unmc.edu

¥M.W. and S.PS. contributed equally to this manuscript.

Supporting Information

File 1: PDF, Supplemental Methods, Supplemental Figures S1-S3.

File 2: CSV: Supplemental Tables S1-S2: Analysis of normalized peak area and percentage relative standard deviation (RSD%) from peak area obtained from Skyline analysis.

Supplemental Methods

Glycan sample preparation

Briefly, serum equivalent to 150 µg protein was reduced, alkylated, neutralized, resuspended in binding buffer then loaded onto each well of the glyPAQ protein capture plate. *N*-glycans were released with PNGase F for 12 hours at 37˚C then reduced per manufacturer's instructions. *N*-glycans were then cleaned using PGC as described[21], then dried and reconstituted in 20 µl LC-MS grade water. The samples were pooled, and neat (undiluted) and diluted (10x, 100x, 1000x, 10,000x with additional LC-MS water) samples were used for PGC-LC MS analysis.

PGC-LC MS

The PGC microcolumn was held at a constant temperature of 65°C and grounded using a metal union and grounding clamp. To avoid mobile phase pH changes which can affect analyte elution time and degrade silica-based parts, a primary stock of 500 mM ammonium bicarbonate, pH 7 was prepared every three days and used to make all mobile phases and both mobile phases were maintained at 4-8 °C in a refrigerator adjacent to the LC. Mobile phase A was comprised of 10 mM ammonium bicarbonate and mobile phase B was 10 mM ammonium bicarbonate in 60% (v/v) acetonitrile. Five µL of sample (equivalent to *N*-glycans released from 40, 4, 0.4, 0.04, 0.004 µg total protein for neat and diluted samples, respectively) were injected directly onto the column with 2 µL/min flow in 2% buffer B for 3 minutes, and this flow rate was maintained for the acquisition. A post-column makeup flow of acetonitrile at 3 µL/min was connected with a tee junction (Figure 1A) as this method has been reported to improve glycan ionization efficiency and stability[22,23]. This makeup flow was redirected to wash the column with methanol at 65 min using a switching valve. For both sources, a capillary voltage of 2800 V was applied and transfer tube temperature of 250 °C at 10 L/min. The HESI source was operated at position 1, sheath gas flow at 10 units, voltage at -2800 V. A multi-step gradient of B: 2–8% in 3 min and 8–35% in 60 min was utilized. MS spectra were acquired with a *m/z* range of 500–2000; AGC target set to 8×10^5 and maximum injection time to 200 ms. Fragments of the most abundant precursors were

generated using collision-induced dissociation at 33%, isolated within 2 ppm, for a maximum scan time of 4 seconds. MS/MS AGC target was set to 2×10^4 and maximum injection time to 200 ms.

MS Data Analysis

Raw data were imported into Skyline-daily (64-bit) 21.1.1.223[24] and structures contained in an in-house curated *N*-glycan transition list for NIST serum containing 210 *N*-glycans were integrated. Peak picking was performed manually. Three isotopic envelopes with a centroid mass accuracy value of 15 ppm were used for peak integration. Structural assignment of precursor mass was performed manually using GlycoWorkBench v2.1[25]. Structural annotation was made based on retention time, characteristic A/X, B/Y, and C/Z product ions in MS/MS scans and elution order described previously[26]. Additionally, diagnostic D ions, D ion-18, D ion-221, and E ions were used to identify glycans moieties linked to the 3-Man and 6-Man arm, bisecting type structures and tri-antennary structures, respectively[13]. Data are available at https://glycopost.glycosmos.org/GPST000359 and in Supporting Information.

To evaluate the effect of ionization source differences on data analysis performed by alternative software used for glycan composition analysis, raw files were also processed using GlycReSoft version 0.4.13[27]. Briefly, a reduced glycan search space containing feasible human *N*-glycans, limited to 26 monosaccharides, was used as provided in the software. Mass matching error tolerance was set to 10 ppm and peak grouping error tolerance was set to 15 ppm. No additional model features were chosen.

Sensitivity of MnESI with M3 emitter normalized to Ion Max NG ion source with ESI emitter (normalized sensitivity) and relative standard deviation were calculated from extracted peak area values of the glycans identified.

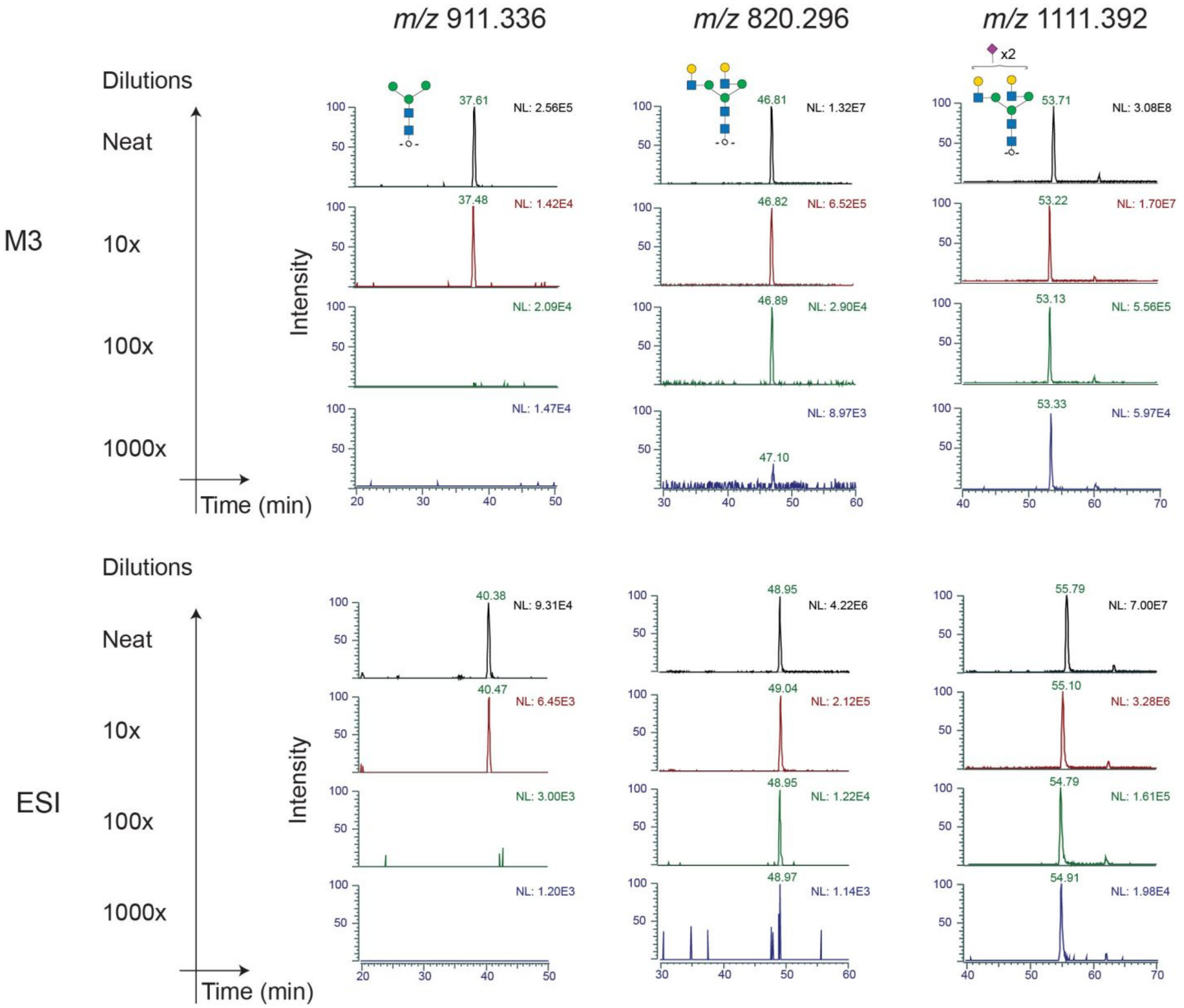

Supporting Figure S1. Extracted ion chromatograms for three *N*-glycans detected in serially diluted samples using M3 or ESI emitter.

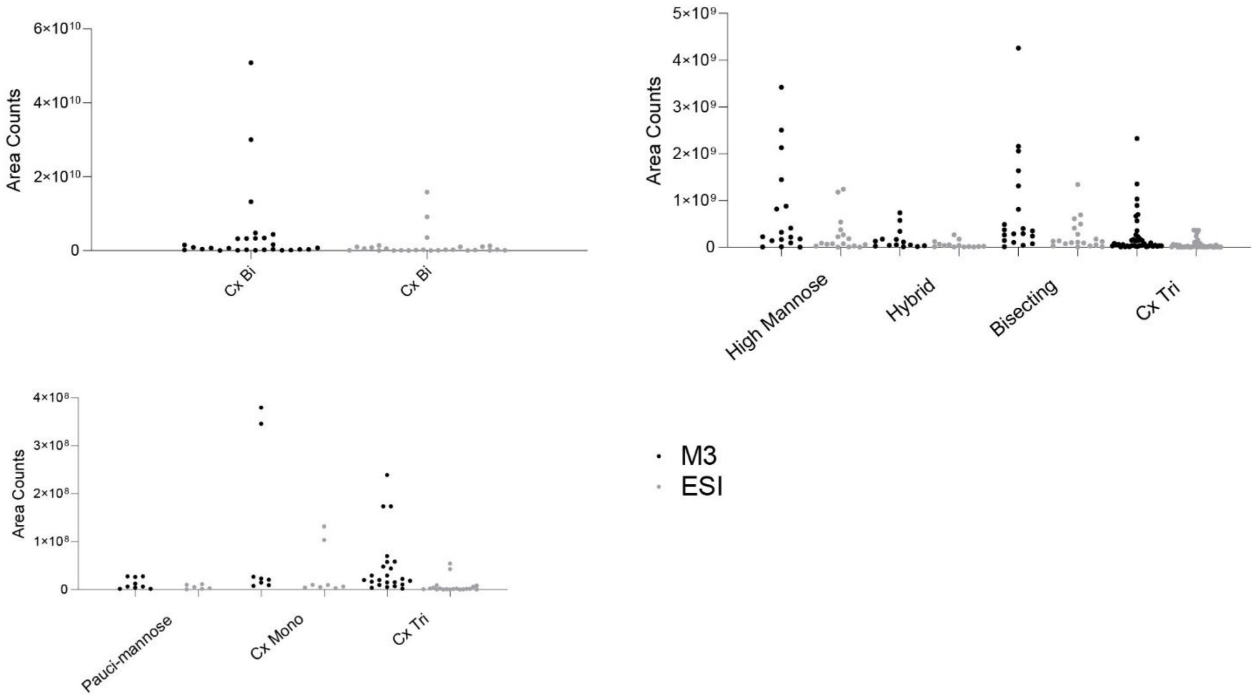

Supporting Figure S2. Raw area counts of individual glycans in each glycan class for three replicate injections using the M3 and ESI. Abbreviations: PM - Paucimannose, HM - High-mannose, CxM - Complex-mannose, CxBi - Complex biantennary, CxTri - Complex Triantennary CxTet - Complex tetraantennary

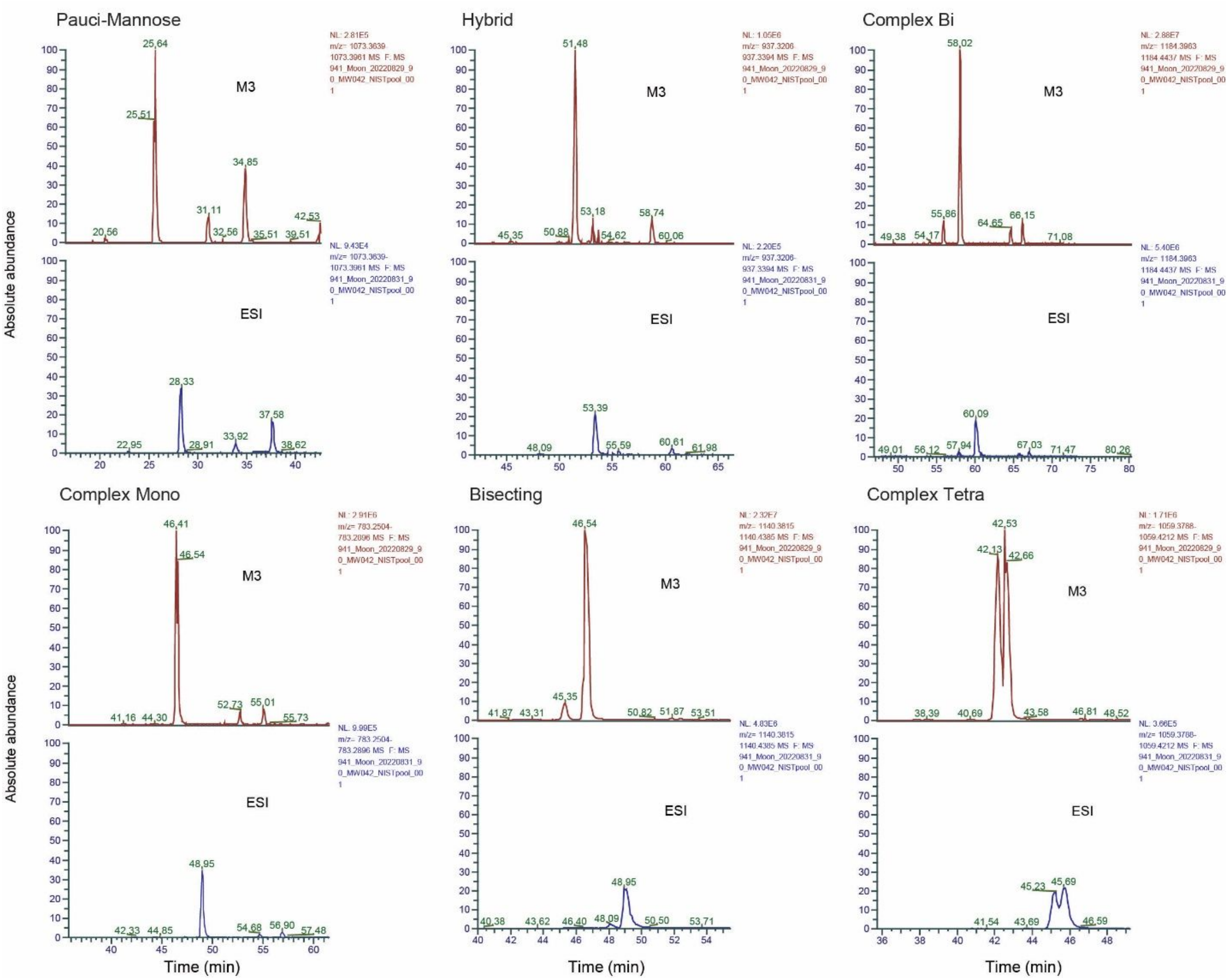

Supporting Figure S3. Extracted ion chromatograms of representative glycan class peaks using the M3 and ESI.
